# A Modification to Two‐Stage Least Squares With Genetic Applications

**DOI:** 10.1002/sim.70308

**Published:** 2025-11-07

**Authors:** Lei Fang, Wei Pan

**Affiliations:** ^1^ Division of Biostatistics and Health Data Science University of Minnesota Minneapolis Minnesota USA

**Keywords:** 2sls, causal inference, instrumental variable regression, r2sls, twas

## Abstract

Two‐stage least squares (2SLS) is by default applied to infer a putative causal association between an exposure, such as a gene or a protein, with an outcome such as a complex disease or trait, in transcriptome‐ or proteome‐wide association studies (TWAS/PWAS). In a typical two‐sample setting for TWAS/PWAS, the stage 1 sample size is much smaller than that of stage 2. To reduce the resulting attenuation bias and estimation uncertainty in stage 1 and boost the statistical power of the conventional TWAS, we propose a new method, called reverse two‐stage least squares (r2SLS): Instead of imputing a gene's expression (using genetic variants as instrumental variables, IVs) in stage 1 and then testing the association between the imputed expression and the observed outcome in stage 2 in the conventional 2SLS approach, we propose predicting the outcome (using IVs) and testing the association between the predicted outcome and the observed gene expression. Theoretically, we establish that the r2SLS estimator is asymptotically unbiased with a normal distribution. We also show theoretically when 2SLS and r2SLS are asymptotically equivalent and when r2SLS is asymptotically more efficient than 2SLS. We also consider the practical issue of how to select invalid IVs. We use simulations and three real data examples based on the GTEx gene expression data, UKB‐PPP proteomic data, and several GWAS summary datasets to demonstrate some advantages of r2SLS over 2SLS, including possibly better type I error control, higher statistical power and robustness to weak IVs.

## 1 Introduction

Genetic studies aim to discover genetic factors associated with disease, facilitating the understanding of the underlying mechanism of disease and thus the development of personalized medicine [1]. In particular, in the last twenty years, genome‐wide association studies (GWAS) have successfully uncovered tens of thousands of genetic variants and loci associated with various complex diseases and traits [2, 3]. However, these GWAS hits usually do not directly offer mechanistic insights [4]. Accordingly, transcriptome‐, proteome‐ and metabolome‐wide association studies (TWAS/PWAS/MWAS) have been proposed to identify (causally) related genes, proteins, and metabolites to shed biological insights [5, 6, 7, 8]; more backgrounds and related references can be found in a recent review [9]. We will use TWAS as a motivating example. In stage 1 of TWAS, one trains a model to predict a gene's expression level using genetic variants like SNPs. In stage 2, one imputes the gene's expression using the genetic variants and tests its association with the outcome. Hence, an interpretation of TWAS is to detect the association between the genetically regulated expression (GREX) of a gene with an outcome [5]. It turns out that the above two‐stage procedure in TWAS is an instance of two‐stage least squares (2SLS) in instrumental variable (IV) regression (with genetic variants as IVs); under suitable conditions (to be elaborated next), TWAS can be interpreted as detecting causal genes for the outcome [6].

In practice, two‐stage least squares (2SLS) is performed in a two‐sample setting for TWAS. Typically, stage 1 is conducted on a relatively small sample, for example, only in hundreds, as in the high‐impact GTEx data we used in our motivating example [10], due to the high cost of collecting gene expression data. On the other hand, stage 2 involves a much larger sample of GWAS on an outcome/trait of interest. In such a practical setting, inference based on 2SLS may be problematic. When the sample size in stage 1 is small, especially when the SNPs/IVs are only weakly associated with the exposure, the exposure would be poorly predicted with a large estimation uncertainty. Consequently, the causal estimate in stage 2 analysis would be biased towards the null, called attenuation bias [11]. In practice, the F‐statistic is often adopted as a criterion (e.g., >10) to check the presence of weak IVs in 2SLS. However, with a small sample size in stage 1, for example, in hundreds, the threshold is difficult to achieve. The consequence is that many exposures (e.g., genes) would be excluded with such a stringent criterion, thereby limiting the discovery of new exposure‐outcome associations. In addition, simply relying on F‐statistics does not guarantee avoidance of weak IV bias [12]. The bias from 2SLS could also further inflate the Type I error [13, 14].

In this report, we develop a novel approach, called reverse two‐stage least squares (r2SLS), by modifying 2SLS to address its issues. Rather than using IVs to predict the exposure based on the stage 1 sample, we leverage the larger sample size in stage 2 to predict the outcome by IVs, then test the association between the predicted outcome and the observed exposure. Intuitively, our prediction step will be more accurate, and so will be the final inference. In particular, our r2SLS also avoids the problem of weak IVs because we do not use the IV‐predicted exposure as a regressor.

The rest of the article is organized as follows: In Section 2, we introduce our model and proposed r2SLS along with its theoretical property and comparison with 2SLS. We also suggest how to handle invalid IVs by incorporating an existing method to identify them. In Section 3, through simulations, we demonstrate the superior performance of r2SLS over 2SLS in point estimation, Type I error control, and statistical power in some scenarios. Finally, in Sections 3.4 and 3.5, we confirm the advantage of r2SLS in two real data applications of TWAS with the GTEx gene expression data, UKB‐PPP proteomic data, and an Alzheimer's disease GWAS summary dataset. In addition, we also show that r2SLS does not introduce false positives in some negative control experiments. We end with a short summary and discussion.

## 2 Methods

Let X∈ℝ be an exposure of interest (e.g., a gene's expression in TWAS), Y∈ℝ be an outcome of interest, and $Z\in {\mathbb{R}}^{p}$ be some genetic variants (i.e., SNPs) to be used as IVs. We consider the most representative two‐sample setting: For simplicity of presentation, we assume that we have individual‐level data in two independent samples, ${𝔻}_{1}=\left\{(x_{1i},Z_{1i})|i=1,\ldots ,n_{1}\right\}$ and ${𝔻}_{2}=\left\{(y_{2i},Z_{2i})|i=1,\ldots ,n_{2}\right\}$; we will extend the method to GWAS summary data later. We use the vector or matrix form $x_{1}\in {\mathbb{R}}^{n_{1}}$, $Z_{1}\in {\mathbb{R}}^{n_{1}\times p}$, $y_{2}\in {\mathbb{R}}^{n_{2}}$, $Z_{2}\in {\mathbb{R}}^{n_{2}\times p}$ to represent the corresponding samples. We also assume that $n_{2}$ is usually much larger than $n_{1}$ with a fixed p.

We consider the following two‐stage structural model:

(1) $$\begin{array}{ll} x_{1} & =Z_{1}\beta +\varepsilon \; \end{array}$$

(2) $$\begin{array}{ll} y_{2} & =x_{2}\theta +\xi ,\; \end{array}$$

where the random errors ε and ξ both follow a normal distribution with mean 0, and are independent of both $Z_{1}$ and $Z_{2}$. These random errors may contain environmental and other confounding effects, and thus are correlated if observed in the same sample. The exposure $x_{2}$ is not observed but assumed to have the same distribution as (1). Similarly, $y_{1}$ is not observed in the stage 1 sample but assumed to follow the same model as (2). Without loss of generality, we assume each IV in $Z_{1}$ and $Z_{2}$, $x_{1}$ and $y_{2}$ are standardized to have mean 0 and standard deviation 1. Our goal is to infer θ, often representing the causal effect of the exposure on the outcome.

### 2.1 New Method: r2SLS

Our strategy is to use a predicted outcome to draw an inference. Since we can rewrite (2) as $y_{2}=Z_{2}\beta \theta +\varepsilon \theta +\xi$, our first step is to estimate βθ by regressing $y_{2}$ on $Z_{2}$ via ordinary least squares (OLS) with sample ${𝔻}_{2}$, obtaining an estimate $\tilde{\beta \theta }$, then predict $y_{1}$ as ${\hat{y}}_{1}=Z_{1}\tilde{\beta \theta }$. Using the working model:

(3) $$\begin{array}{l} {\hat{y}}_{1}=x_{1}\theta +\varepsilon_{y_{1}}^{\prime }, \end{array}$$

with $\varepsilon_{y_{1}}^{\prime }=Z_{1}(\tilde{\beta \theta }-\beta \theta )-\varepsilon \theta ,$ we study the OLS estimator $\hat{\theta }$ and derive its distribution. Specifically, 

(4)

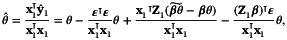


where $\hat{\theta }$ can be decomposed into four parts: The true θ, a bias component that depends on the true θ, and two random terms that are from stage 2 and stage 1 estimation, respectively.

Under the null hypothesis θ=0, $\hat{\theta }$ is an unbiased estimator. Thus, we have the following lemma:

Lemma 1 *Under models* (*1*) *and* (*2*), *when* $Z_{2}^{⊺}Z_{2}/n_{2}$ *is invertible, and* $\hat{\theta }$ *is defined in Equation* (*4*), *then under the null hypothesis* θ=0, *we have the distribution of* $\hat{\theta }$ *as follows*:

(5) $$\begin{array}{l} \sqrt{n_{2}}\hat{\theta }∼N(0,\sigma_{t}^{2}\Phi \Psi \Phi^{⊺}), \end{array}$$

*where* $\Phi =x_{1}^{⊺}Z_{1}/x_{1}^{⊺}x_{1},\Psi ={\left(Z_{2}^{⊺}Z_{2}/n_{2}\right)}^{-1},$ *and* $\sigma_{t}^{2}$ represents the total variance of εθ+ξ. *If* $n_{1},n_{2}\to \infty$, *then* $\Phi =\lim_{n_{1}\to \infty }x_{1}^{⊺}Z_{1}/x_{1}^{⊺}x_{1}=\beta^{⊺}\sum_{Z}$ *and* $\Psi =\lim_{n_{2}\to \infty }{\left(Z_{2}^{⊺}Z_{2}/n_{2}\right)}^{-1}=\sum_{Z}^{-1}$. *We have*

(6) $$\begin{array}{l} \sqrt{n_{2}}\hat{\theta }\overset{d}{\to }N(0,\sigma_{t}^{2}\beta^{⊺}\sum_{Z}\beta ). \end{array}$$

Remark 1 A naive method for drawing inference on θ with $\hat{\theta }$ is to estimate the standard error from the OLS using $\mathrm{var}({\hat{y}}_{1})/(n_{1}\mathrm{var}(x_{1}))$. However, as shown in Section 3, it is incorrect, leading to inflated Type I errors. The reason is that $\varepsilon_{y_{1i}}^{\prime }$'s are no longer identically distributed. Specifically, for each observation i, we have $Z_{1i}(\tilde{\beta \theta }-\beta \theta )∼N(0,\sigma_{t}^{2}Z_{1i}{\left[Z_{2}^{⊺}Z_{2}\right]}^{-1}Z_{1i}^{⊺})$. Since $Z_{1i}$'s are different across i's, the variances of $\varepsilon_{y_{1i}}^{\prime }$'s are not equal. We also tried weighted least squares (WLS) to handle this heteroskedasticity problem in the simulation study. Unfortunately, it did not work well as expected.

When θ≠0, there are two additional components in $\hat{\theta }$: $\varepsilon^{⊺}\varepsilon \theta /x_{1}^{⊺}x_{1}$ and ${\left(Z_{1}\beta \right)}^{⊺}\varepsilon \theta /x_{1}^{⊺}x_{1}$. Since $E(\varepsilon^{⊺}\varepsilon \theta /x_{1}^{⊺}x_{1})\neq 0$ and $E({\left(Z_{1}\beta \right)}^{⊺}\varepsilon \theta /x_{1}^{⊺}x_{1})=0$, $\hat{\theta }$ is a biased estimate and the bias exclusively results from $\varepsilon^{⊺}\varepsilon \theta /x_{1}^{⊺}x_{1}$. Denotes the bias factor $\kappa =\varepsilon^{⊺}\varepsilon /x_{1}^{⊺}x_{1}=1-R^{2}$, where $R^{2}$ is the (empirical/sample) multiple coefficient of determination for the stage 1 model (1). Based on (4), to obtain an unbiased estimate of a non‐zero θ, we define a new estimator $\tilde{\theta }=\hat{\theta }/(1-\kappa )$. Then by incorporating the additional variance component ${\left(Z_{1}\beta \right)}^{⊺}\varepsilon \theta /x_{1}^{⊺}x_{1}$, the distribution of $\tilde{\theta }$ is given in Theorem 1.

Theorem 1 *Under models* (*1*) *and* (*2*), *when* $Z_{2}^{⊺}Z_{2}/n_{2}$ *is invertible, we have the distribution of* $\tilde{\theta }$ *as follows*:

(7) $$\begin{array}{c} \sqrt{n_{2}}(\tilde{\theta }-\theta )∼N\left(0,\frac{1}{{\left(1-\kappa \right)}^{2}}(\sigma_{t}^{2}\Phi \Psi \Phi^{⊺}+n_{2}\theta^{2}\sigma_{\varepsilon }^{2}\|Z_{1}\beta {\|}_{2}^{2}/{\left(x_{1}^{⊺}x_{1}\right)}^{2})\right), \end{array}$$

*where* $\sigma_{\varepsilon }^{2}$ *is the variance of* ε *and* 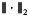 *is the L_2_ norm. If* $n_{1},n_{2}\to \infty$ *and* $\lim_{n_{1},n_{2}\to \infty }\;n_{2}/n_{1}=\phi \in (0,\infty )$, *then we have*

(8) $$\begin{array}{l} \sqrt{n_{2}}(\tilde{\theta }-\theta )\overset{d}{\to }N\left(0,\sigma_{t}^{2}/(\beta^{⊺}\sum_{Z}\beta )+\phi \theta^{2}\sigma_{\varepsilon }^{2}/(\beta^{⊺}\sum_{Z}\beta )\right). \end{array}$$

Remark 2 In practice, we recommend using (7) to conduct hypothesis testing if the stage 1 sample size $n_{1}$ is small. The variance component $n_{2}\theta^{2}\sigma_{\varepsilon }^{2}\|Z_{1}\beta {\|}_{2}^{2}/{\left(x_{1}^{⊺}x_{1}\right)}^{2}$ can be estimated as $\left(n_{2}/n_{1})\tilde{\theta }\kappa (1-\kappa \right)$.

As $n_{1}\to \infty$, 1−κ converges to the (population) coefficient of determination $R^{2}$. To estimate 1−κ, we propose applying either the Olkin‐Pratt $R^{2}$ estimator [15] or the adjusted $R^{2}$. Both methods provide similar performance in most cases. In general, we also do not recommend using unadjusted $R^{2}$ since it is biased, as shown in Table S1. When $R^{2}$ is low, for example the expression levels of some genes are not highly heritable, either the Olkin‐Pratt or the adjusted $R^{2}$ estimate could be 0 or even negative, in which case the bias correction in r2SLS is not applicable. Therefore, in practice, if $R^{2}$ in stage 1 is too small, for example, <0.01, we recommend using (5) directly to test null hypothesis $H_{0}:\theta =0$. See Table S2 for some simulation results in such a case.

When individual‐level data are not available, our method still works with GWAS summary data. Specifically, we use the adjusted $R^{2}$ (to estimate $R^{2}$ as the Olkin‐Pratt estimator is not applicable to summary statistics). The estimate $\hat{\theta }$ is calculated as $\text{cor}(x_{1},Z_{1})\tilde{\beta \theta }$, where $\text{cor}(x_{1},Z_{1})$ is the vector of correlations of each SNP in Z with exposure X calculated from the GWAS summary statistics. Specifically, for any SNP $Z_{j}$, given the marginal estimate of its effect size ${\hat{\gamma }}_{jg}$ and variance $\mathrm{var}({\hat{\gamma }}_{jg})$, the marginal correlation $r_{jg}$ is calculated [16] as

$$\begin{array}{l} r_{jg}=\frac{{\hat{\gamma }}_{jg}}{\sqrt{{\hat{\gamma }}_{jg}^{2}+(N_{j}-2)\cdot \mathrm{var}({\hat{\gamma }}_{jg})}}, \end{array}$$

where $N_{j}$ is the corresponding sample size. For the standard error, Ψ is the inverse of the correlation matrix of Z that can be estimated from a reference panel. And the other components can also be estimated using their sample versions.

### 2.2 Comparison With 2SLS

The conventional 2SLS method uses the OLS in the stage 1 model (1) to predict the exposure in stage 2 (2) for inference. From [17], we first have

$$\begin{array}{ll} \sqrt{n_{1}}(\hat{\beta }-\beta ) & \overset{d}{\to }N(0,\sigma_{\varepsilon }^{2}/\sum_{Z}) \end{array}$$

as $n_{1}\to \infty$. Then the predicted ${\hat{x}}_{2}=Z_{2}\hat{\beta }$, and the estimator for θ is 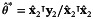. The asymptotic distribution for ${\hat{\theta }}^{*}$ as $n_{2}\to \infty$ is

(9) $$\begin{array}{l} \sqrt{n_{2}}({\hat{\theta }}^{*}-\theta )\overset{d}{\to }N(0,\sigma_{t}^{2}/\beta^{⊺}\sum_{Z}\beta +\phi \theta^{2}\sigma_{\varepsilon }^{2}/\beta^{⊺}\sum_{Z}\beta ). \end{array}$$

From Theorem 1, the asymptotic variance of $\tilde{\theta }$ is the same as ${\hat{\theta }}^{*}$, thus r2SLS reaches the same asymptotic efficiency as 2SLS. However, with finite samples, especially when the stage 1 sample size is small and the signals of IVs are weak, the accuracy of β is not attainable, leading to poor prediction of ${\hat{x}}_{2}$. The resulting 2SLS estimator ${\hat{\theta }}^{*}$ would bias the θ toward zero, as a well‐known phenomenon of attenuation in the measurement error literature. This scenario was discovered before [18] and further discussed in the context of genetic studies [14, 19]. As demonstrated in the simulation in Section 3, this bias could inflate Type I errors. On the contrary, r2SLS leverages the large sample size in stage 2 to yield a good prediction of ${\hat{y}}_{1}$, largely alleviating the corresponding attenuation bias.

It is notable that the above asymptotic equivalence between 2SLS and r2SLS is achieved when the same set of SNPs/IVs is used to impute the exposure in 2SLS and to impute the outcome in r2SLS. On the other hand, in TWAS applications of 2SLS, the standard way to select IVs is to use some cis‐SNPs that are located around a gene's coding region. However, most gene expression variation is regulated by trans‐SNPs far away from the gene and dispersed across the whole genome [20]. Due to the small sample size in stage 1, trans‐SNPs are usually not used in 2SLS/TWAS. However, for r2SLS, the large stage 2 sample size allows trans‐SNPs to be considered for predicting ${\hat{y}}_{1}$ and potentially increase power. With additional trans‐SNPs for prediction, we would have a smaller asymptotic variance of $\tilde{\theta }$ as demonstrated in Corollary 1 below. Thus, in these situations, r2SLS would be (asymptotically) *moreefficient* than 2SLS. The corresponding advantage of r2SLS with higher power than 2SLS will be confirmed in simulations.

Corollary 1 *In the true model* (*1*), *we decompose* $Z_{1}=(Z_{1,\text{cis}},Z_{1,\text{trans}})$ *and thus have*

(10) $$\begin{array}{ll} x_{1} & =Z_{1,\text{cis}}\beta_{\text{cis}}+Z_{1,\text{trans}}\beta_{\text{trans}}+\varepsilon . \end{array}$$

*In addition, we assume* $Z_{1,\text{cis}}$ *is independent of* $Z_{1,\text{trans}}$. *If we only use* $Z_{1,\text{cis}}$ *to predict* ${\hat{x}}_{1}$ *as in a typical application of* 2*SLS to TWAS, obtaining the corresponding estimator* ${\hat{\theta }}_{\text{cis}}^{*}$ *of* θ, *we have*

(11) $$\begin{array}{l} \sqrt{n_{2}}({\hat{\theta }}_{\text{cis}}^{*}-\theta )\overset{d}{\to }N(0,\sigma_{t}^{2}/\beta_{\text{cis}}^{⊺}\sum_{Z_{\text{cis}}}\beta_{\text{cis}}+\phi \theta^{2}\sigma_{\varepsilon }^{2}/\beta_{\text{cis}}^{⊺}\sum_{Z_{\text{cis}}}\beta_{\text{cis}}). \end{array}$$

Remark 3 It is clear that we have $\beta_{\text{cis}}^{⊺}\sum_{Z_{\text{cis}}}\beta_{\text{cis}}\leq \beta^{⊺}\sum_{Z}\beta$, thus the variance in Equation (11) is no smaller than that in Equation (8).

### 2.3 Invalid IVs

In models (1) and (2), we assume that all SNPs are valid IVs, satisfying the following IV assumptions [11]: A valid IV is

1. associated with the exposure;
2. independent of the hidden confounders of the exposure‐outcome association;
3. independent of the outcome, conditional on the exposure and the hidden confounders.

The second IV assumption also implies that the instrument‐outcome relationship should not be confounded. However, in genetic studies, there is a widespread presence of the pleiotropic effect of SNPs [21], meaning that SNPs may affect the outcome directly or through other hidden confounders. Here, we consider the situation when there are invalid IVs. In this situation, model (2) becomes the following model

(12) $$\begin{array}{ll} y_{2} & =x_{2}\theta +Z_{2}\alpha +\xi \; \end{array}$$

to adjust such a pleiotropic effect. The support of α represents those invalid IVs. To obtain a correct inference of θ in the presence of invalid IVs, we adopted a two‐step procedure. The first step is to use TScML [21] to select those invalid IVs, which is defined as a set $\hat{B}$ while the true set is B. The key advantage of TScML is its ability to consistently identify the invalid IVs with a theoretical guarantee. Then in the second step, we adjust the effect of those identified invalid IVs in step 1, to infer θ by considering the following working model:

(13) $$\begin{array}{l} {\hat{y}}_{1}=x_{1}\theta +Z_{1,B}\alpha_{B}+\varepsilon_{y_{1}}^{\prime }. \end{array}$$

Define $\hat{\theta^{\prime }}=\frac{x_{1}(I-P_{Z_{1,\hat{B}}}){\hat{y}}_{1}}{x_{1}^{⊺}(I-P_{Z_{1,\hat{B}}})x_{1}}$ with P  represents the projection matrix, then our proposed r2SLS with invalid selection (r2SLS‐S) estimator $\tilde{\theta^{\prime }}=\hat{\theta^{\prime }}/(1-\kappa^{\prime })$, where $\kappa^{\prime }=\frac{\varepsilon^{⊺}\varepsilon (1-m/n_{1})}{x_{1}^{⊺}(I-P_{Z_{1,\hat{B}}})x_{1}}$, where $m=|\hat{B}{|}_{0}$. Let $\lim_{n_{1}\to \infty }\frac{\sqrt{n_{1}}\|Z_{1,{\hat{B}}^{c}}\beta_{{\hat{B}}^{c}}(I-P_{Z_{1,\hat{B}}}){\|}_{2}}{x_{1}^{⊺}(I-P_{Z_{1,\hat{B}}})x_{1}}=\tau$, $\lim_{n_{1}\to \infty }\frac{x_{1}^{⊺}(I-P_{Z_{1,\hat{B}}})Z_{1}}{x_{1}^{⊺}(I-P_{Z_{1,\hat{B}}})x_{1}}=\Phi^{\prime }$, we then have the following theorem.

Theorem 2 *Under models* (*1*) *and* (*12*), *when* $Z_{2}^{⊺}Z_{2}/n_{2}$ *is invertible and other mild conditions, in addition*, $n_{1},n_{2}\to \infty$ *and* $\lim_{n_{1},n_{2}\to \infty }\;n_{2}/n_{1}=\phi \in (0,\infty )$, *TScML selects the true invalid set B with probability converges to* 1. *We then have the following asymptotic distribution of* ${\tilde{\theta }}^{\prime }$:

(14) $$\begin{array}{l} \sqrt{n_{2}}({\tilde{\theta }}^{\prime }-\theta )\overset{d}{\to }N\left(0,\frac{1}{{\left(1-\kappa^{\prime }\right)}^{2}}(\sigma_{t}^{2}\Phi^{\prime }\Psi \Phi^{\prime ⊺}+\phi \theta^{2}\sigma_{\varepsilon }^{2}\tau^{2})\right). \end{array}$$

Remark 4 The additional mild conditions include the plurality condition and eigenvalue conditions of the covariance matrix of $Z_{2}$ for TScML to have a variable selection consistency, see the supplement for the details.

Remark 5 Ideally, we would want to develop an invalid IV selection method (similar to TScML) under our r2SLS framework. However, due to the fact that conditional on $Z_{1}$, $x_{1}$ is independent of ${\hat{y}}_{1}$, because, by construction, $Z_{1}$ is directly causal to ${\hat{y}}_{1}$, not mediated through $x_{1}$. Thus, it is challenging to select those invalid IVs under the r2SLS framework; we leave this as a future topic.

Remark 6 In practice, when selecting IVs for r2SLS, we recommend including both IVs (e.g., cis‐SNPs) related to the (gene/protein) exposure (based on the exposure GWAS data) and IVs (e.g., trans‐SNPs) associated with the outcome (based on the outcome GWAS data). There are two reasons to include IVs that are related to the exposure. First, selecting IVs based solely on their association with the outcome can be problematic: If none of these IVs are related to the exposure, then all IVs are invalid. Moreover, if most IVs are unrelated to the exposure, the predicted outcome will contain substantial variability unrelated to the exposure, which increases the estimation variance and thus reduces power. At the same time, including IVs related to the outcome, which may be trans‐SNPs for the exposure, may improve statistical power, especially with a small dataset of exposure. On the other hand, with a large dataset of exposure, using IVs only related to the exposure may have sufficient power.

### 2.4 Extension to Multiple Exposures

We extend r2SLS to multivariate analysis with multiple exposures. Suppose we have d exposures of interest, a typical multivariate two‐stage model in the two‐sample setting is the following:

(15) $$x_{1,j}=Z_{1}\beta_{j}+\varepsilon_{j},\;y_{2}=\sum_{j=1}^{d}x_{2,j}\theta_{j}+\xi ,$$

where $x_{1,j}$ represents exposure j in the stage 1 sample and $y_{2}$ is from the stage 2 sample; all the error terms $\varepsilon_{j}$ and ξ are assumed to be from a multivariate normal distribution.

Denote $X=(x_{1,1},\ldots ,x_{1,d})$, $B=(\beta_{1},\ldots ,\beta_{d})$, $V=(\varepsilon_{1},\ldots ,\varepsilon_{d})$, and $\theta ={\left(\theta_{1},\ldots ,\theta_{d}\right)}^{⊺}$. As in the univariate case, we use $Z_{1}$ to predict ${\hat{y}}_{1}$ as $Z_{1}\hat{\gamma }$. We still use the OLS estimator to derive the corresponding estimator and its asymptotic normality. Specifically, we have $\hat{\theta }={\left(X^{⊺}X\right)}^{-1}X^{⊺}{\hat{y}}_{1}$. Let the correction matrix $\Lambda ={\left\{I_{d}-E\left({\left(X^{⊺}X\right)}^{-1}V^{⊺}V\right)\right\}}^{-1}$, then the unbiased estimator for θ is $\tilde{\theta }=\Lambda \hat{\theta }$. Let $\sigma_{t}^{2}$ denote the total variance of $\sum_{j=1}^{d}\varepsilon_{j}\theta_{j}+\xi$, and $\sigma_{t_{X}}^{2}$ denote the total variance of $\sum_{j=1}^{d}\varepsilon_{j}\theta_{j}$. We have the following asymptotic distribution for $\tilde{\theta }$.

Theorem 3 *Under the model setting* (*15*), *when* $n_{1},n_{2}\to \infty$, *we have*

(16) $$\begin{array}{ll} \sqrt{n_{1}}(\tilde{\theta }-\theta ) & \overset{d}{\to }N(0,\phi \sigma_{t}^{2}\Lambda W^{⊺}E{\left(Z_{1}^{⊺}Z_{1}\right)}^{-1}W\Lambda \\ & \;+\sigma_{t_{X}}^{2}\Lambda {\left(\sum_{X}\right)}^{-1}B^{⊺}\sum_{Z}B{\left(\sum_{X}\right)}^{-1}) \end{array}$$

*with* $W=E{\left(X^{⊺}X\right)}^{-1}E(X^{⊺}Z_{1})$.

Λ can be estimated as 
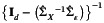
, where each entry in ${\hat{\sum }}_{X}$ is the sample covariance of $x_{1,i}$ and $x_{1,j}$, and each entry in ${\hat{\sum }}_{\varepsilon }$ is the sample covariance of $\varepsilon_{i}$ and $\varepsilon_{j}$.

We can also consider the presence of invalid IVs in the multivariate case using the same strategy as before, that is, selecting invalid IVs via a constrained maximum likelihood approach [22].

## 3 Results

### 3.1 Simulations: More Accurate Estimation and Better Controlled Type I Errors by r2SLS

We conducted simulations to investigate the operating characteristics of r2SLS as compared with the following methods: r2SLS, r2SLS‐naive, 2SLS, observed‐1, observed‐2, and weighted least squares (WLS). In r2SLS we used the adjusted $R^{2}$ to calculate κ. The method r2SLS‐naive used the predicted ${\hat{y}}_{1}$ to conduct a simple linear regression on the observed exposure with the stage 1 sample. Both observed‐1 and observed‐2 performed a simple linear regression on the observed exposure and outcome in samples 1 and 2, respectively (while assuming the availability of both the exposure and outcome in both samples). We added WLS as a comparison; the weights were estimated from the squared residuals plus $10^{-6}$ to avoid a possible (near) singularity. Considering a typical TWAS application, we set the stage 1 sample size as $n_{1}=500$. We generated p=30 cis‐SNPs independently from a standard normal distribution with their effects $\beta_{j}∼N(0.1,0.1^{2})$ for j=1,…,30. The random errors ε and ξ in the same sample were drawn from a bivariate normal distribution with mean 0 and a covariance matrix with all non‐diagonals as 0.25 and diagonals as 1. We set the stage 1 $R^{2}=0.235$. We also considered a weak IV scenario with p=120 cis‐SNPs of weaker effects $\beta_{j}∼0.5*N(0.1,0.1^{2})$ but with the same $R^{2}$. The stage 2 sample size was $n_{2}=10\;000$ with θ∈{0,0.1,0.2}. The results for estimating θ are summarized in Table 1 based on 500 repetitions in each scenario.

**TABLE 1:** Simulation results for estimating θ and testing $H_{0}:\theta =0$ versus $H_{0}:\theta \neq 0$ for each method with $n_{1}=500$ and $n_{2}=10\;000$. SD, SE, and MSE represent the standard deviation of the estimates, their mean standard error, and mean squared error, respectively.

		θ=0					θ=0.1					θ=0.2				
	Methods	Mean	SD	SE	MSE	Type‐I	Mean	SD	SE	MSE	Power	Mean	SD	SE	MSE	Power
p=30	r2SLS	1.32e−4	0.020	0.021	4.03e−4	0.042	0.102	0.022	0.023	4.96e−4	0.996	0.204	0.028	0.028	8.08e−4	1.000
$\beta_{j}∼N(0.1,0.1^{2})$	2SLS	‐7.86e−5	0.016	0.017	2.58e−4	0.040	0.083	0.017	0.018	5.60e−4	0.998	0.167	0.020	0.022	1.47e−3	1.000
	r2SLS‐naive	7.79e−5	0.005	2.11e−3	2.12e−5	0.372	0.023	0.005	2.80e−3	5.89e−3	0.996	0.047	0.006	4.31e−3	0.024	1.000
	observed‐1	0.188	0.038	0.037	0.037	1.000	0.288	0.038	0.038	0.037	1.000	0.388	0.038	0.038	0.037	1.000
	observed‐2	0.190	0.009	0.009	0.036	1.000	0.291	0.009	0.009	0.036	1.000	0.391	0.009	0.009	0.036	1.000
	WLS	5.71e−5	0.005	2.69e−4	2.05e−5	0.892	0.022	0.005	3.19e−4	6.07e−3	1.000	0.044	0.006	4.06e−4	0.024	1.000
p=120	r2SLS	1.21e−3	0.027	0.027	7.35e−4	0.042	0.104	0.031	0.029	9.55e−4	0.976	0.207	0.041	0.033	1.72e−3	1.000
$\beta_{j}∼0.5*N(0.1,0.1^{2})$	2SLS	8.08e−4	0.013	0.013	1.66e−4	0.042	0.051	0.014	0.014	2.62e−3	0.974	0.100	0.016	0.015	0.010	1.000
	r2SLS‐naive	3.87e−4	0.006	4.29e−3	3.59e−5	0.162	0.024	0.007	4.80e−3	5.81e−3	1.000	0.048	0.008	5.94e−3	0.023	1.000
	observed‐1	0.189	0.038	0.038	0.037	1.000	0.289	0.038	0.038	0.037	1.000	0.389	0.038	0.038	0.037	1.000
	observed‐2	0.191	0.009	0.009	0.036	1.000	0.291	0.009	0.009	0.036	1.000	0.391	0.009	0.009	0.036	1.000
WLS	3.63e−4	0.006	4.14e−3	3.62e−5	0.894	0.024	0.007	4.78e−4	5.87e−3	1.000	0.047	0.008	4.91e−3	0.023	1.000

When θ=0, r2SLS performed similarly to 2SLS with well‐controlled Type I errors and high power in both situations, while the other methods, r2SLS‐naive, observed‐1, and observed‐2, were unable to control the Type I error at all. However, if θ≠0, 2SLS suffered from biased estimation, and it became worse with weaker IVs in stage 1 (though the $R^{2}$ in stage 1 remained the same). As θ increased, r2SLS demonstrated a greater advantage by achieving a smaller mean squared error (MSE). In addition, WLS performed badly in this situation. We suspect that because of the difficulty of accurately estimating the weights in the small sample size, the standard error from WLS was not well estimated to obtain a valid inference of θ.

When we used correlated IVs with an autocorrelation of 0.5 while keeping every other aspect of the settings the same, as shown in Table 2, we still reached a similar conclusion. Therefore, r2SLS worked well with correlated IVs.

**TABLE 2:** Simulation results for estimating θ and testing $H_{0}:\theta =0$ versus $H_{0}:\theta \neq 0$ for each method with $n_{1}=500$ and $n_{2}=10\;000$ with IVs of autocorrelation of 0.5. SD, SE, and MSE represent the standard deviation of the estimates, their mean standard error, and mean squared error, respectively.

		θ=0					θ=0.1					θ=0.2				
	Methods	Mean	SD	SE	MSE	Type‐I	Mean	SD	SE	MSE	Power	Mean	SD	SE	MSE	Power
p=30	r2SLS	1.01e−4	0.011	0.011	1.32e−4	0.046	0.101	0.013	0.013	1.65e−4	1.000	0.201	0.016	0.022	2.53e−4	1.000
$\beta_{j}∼N(0.1,0.1^{2})$	2SLS	‐1.57e−4	0.011	0.010	1.13e−4	0.046	0.094	0.012	0.012	1.77e−4	1.000	0.187	0.014	0.014	3.59e−4	1.000
p=120	r2SLS	5.80e−5	0.013	0.013	1.73e−4	0.048	0.101	0.015	0.014	2.12e−4	1.000	0.202	0.018	0.014	3.32e−4	1.000
$\beta_{j}∼0.5*N(0.1,0.1^{2})$	2SLS	5.34e−5	0.006	0.004	3.78e−5	0.054	0.075	0.010	0.010	7.49e−4	1.000	0.149	0.012	0.012	2.7e−3	1.000

**FIGURE 1:**
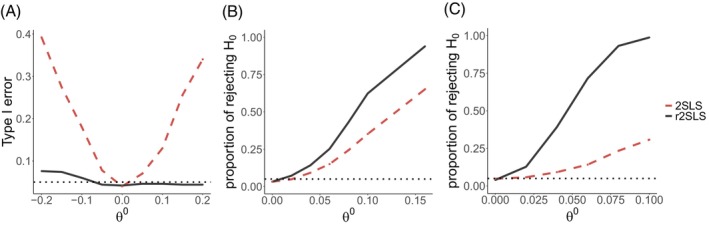
Comparison of r2SLS and 2SLS in simulations: (A) Type I errors for r2SLS and 2SLS for testing $H_{0}:\theta =\theta^{0}$ versus $H_{A}:\theta \neq \theta^{0}$ with 30 cis‐SNPs; (B) Type I errors (for $\theta^{0}=0$) and power (for $\theta^{0}\neq 0$) for r2SLS and 2SLS for testing $H_{0}:\theta =0$ versus $H_{A}:\theta \neq 0$ with 20 trans‐SNPs; (C) Similar to (B) but with 20 trans‐SNPs of stronger effects. The horizontal dashed line represents the nominal significance level of 0.05.

Furthermore, the bias from 2SLS also inflated its Type I error when we were testing the null hypothesis $H_{0}:\theta =\theta^{0}$ versus $H_{A}:\theta \neq \theta^{0}$ for a non‐zero $\theta^{0}$. As Figure 1A shows, the Type I error was not well controlled by 2SLS and jumped up quickly as θ increased, while r2SLS yielded much more stable and better controlled Type I errors. When θ became large, r2SLS had a slightly inflated type I error due to the bias from estimating 1/(1−κ) or $1/R^{2}$. The nonlinear nature of this bias correction factor with respect to $R^{2}$ poses a difficulty in obtaining an unbiased estimate, which we leave as future work.

In addition, as shown in Supporting Information: Table S2, if $R^{2}$ was equal or close to 0 in the (true) stage 1 model, unbiased estimation was not feasible by either r2SLS (because the correction factor $1/R^{2}$ would be exactly or nearly ∞) or 2SLS; however, based on (5), one may still use the un‐corrected version of r2SLS to conduct hypothesis testing.

### 3.2 Simulations: r2SLS May Have Higher Power Than 2SLS

To mimic real TWAS applications, we considered both cis‐SNPs and trans‐SNPs. We simulated cis‐SNPs and trans‐SNPs independently for a single exposure with a normal distribution. We had $p_{1}=20$ cis‐SNPs and $p_{2}=20$ trans‐SNPs. We generated the exposure and outcome from the following model:

(17) $$X=\overset{p_{1}}{\underset{j=1}{\sum }}Z_{j}\beta_{j}+\overset{p_{1}+p_{2}}{\underset{j=p_{1}+1}{\sum }}Z_{j}\beta_{j}+\epsilon_{x},\;Y=X\theta +\epsilon_{y}.$$

The errors $\epsilon_{x}$ and $\epsilon_{y}$ were drawn from a bivariate normal distribution with mean 0 and a covariance matrix of non‐diagonals as 0.25 and diagonals as 1. We generated $\beta_{j}$'s from $N(0.05,0.05^{2})$ for cis‐SNPs with j=1,…,20, and from $N(0.05,0.1^{2})$ or $N(0.1,0.1^{2})$ for trans‐SNPs with j=21,…,40. We set the stage 1 and stage 2 sample sizes as $n_{1}=500$ and $n_{2}=10\;000$, respectively. For 2SLS, we only used cis‐SNPs to predict X with the stage 1 sample, whereas for r2SLS, we used both cis‐SNPs and trans‐SNPs to predict Y with the stage 2 sample. With their empirical Type I errors and power in Figure 1B,C based on 500 repetitions, it is clear that r2SLS with trans‐SNPs achieved higher power than 2SLS, and the advantage appeared larger with more variations of the exposure explained by trans‐SNPs.

Finally, when the outcome did not follow a normal distribution, but a skewed or a heavy‐tailed distribution such as an exponential distribution or a t‐distribution with degrees of freedom 5, respectively, r2SLS still outperformed 2SLS with better controlled type I errors and higher power as demonstrated in Supporting Information: Figures S1 and S2, respectively.

### 3.3 Simulations: r2SLS‐S Performed Well in the Presence of Invalid IVs

We evaluated the performance of r2SLS‐S in the presence of invalid IVs. We set the IVs as correlated with an autocorrelation of 0.5, and invalid IVs with $\beta_{j}=0$ or $\beta_{j}∼N(0.1,0.1^{2})$ and $\alpha_{j}$ following Unif(0.1,0.2) for j=1,…,5 when p=30, and for j=1,…,20 when p=120. The rest of the parameters were set the same as in the valid IV case in Section 3.1. In Tables 3 and 4, both 2SLS and r2SLS gave biased estimates, causing inflated Type I errors. In contrast, the proposed r2SLS‐S controlled the Type I error well as TScML, while achieving similar power. This suggests that if we adopt an existing method that can effectively select out invalid IVs, r2SLS‐S would work well in the presence of invalid IVs.

**TABLE 3:** Simulation results with invalid IVs for estimating θ and testing $H_{0}:\theta =0$ versus $H_{0}:\theta \neq 0$ for each method with $n_{1}=500$ and $n_{2}=10\;000$. The invalid IVs $\beta_{j}=0$ and $\alpha_{j}∼\text{Unif}(0.1,0.2)$ for j=1,…,5 when p=30 and for j=1,…,20 when p=120. For the valid IVs, $\beta_{j}∼N(0.1,0.1^{2})$ when p=30, and $\beta_{j}∼0.5*N(0.1,0.1^{2})$ when p=120. SD, SE, and MSE represent the standard deviation of the estimates, their mean standard error, and mean squared error, respectively.

		θ=0					θ=0.025					θ=0.05				
	Methods	Mean	SD	SE	MSE	Type‐I	Mean	SD	SE	MSE	Power	Mean	SD	SE	MSE	Power
p=30	r2SLS‐S	−1.60e−4	0.014	0.013	1.85e−4	0.058	0.025	0.014	0.013	1.90e−4	0.480	0.050	0.014	0.014	2.05e−4	0.960
	r2SLS	0.045	0.043	0.014	3.91e−3	0.698	0.071	0.043	0.014	3.94e−3	0.866	0.096	0.043	0.014	3.98e−3	0.950
	TScML	−1.45e−4	0.013	0.012	1.58e−4	0.054	0.023	0.013	0.012	1.65e−4	0.500	0.046	0.013	0.012	1.87e−4	0.962
	2SLS	0.040	0.033	0.012	2.65e−3	0.716	0.063	0.033	0.012	2.51e−3	0.888	0.086	0.033	0.013	2.37e−3	0.974
p=120	r2SLS‐S	4.91e−4	0.015	0.014	2.23e−4	0.054	0.025	0.015	0.015	2.27e−4	0.414	0.050	0.015	0.015	2.37e−4	0.920
	r2SLS	0.023	0.095	0.016	9.57e−3	0.744	0.048	0.095	0.016	9.61e−3	0.800	0.074	0.095	0.016	9.66e−3	0.838
	TScML	4.67e−4	0.011	0.010	1.15e−4	0.056	0.019	0.011	0.010	1.55e−4	0.466	0.037	0.011	0.010	2.85e−4	0.946
	2SLS	0.016	0.059	0.010	3.75e−3	0.754	0.034	0.059	0.010	3.58e−3	0.796	0.051	0.059	0.011	3.52e−3	0.842

**TABLE 4:** Simulation results with invalid IVs for estimating θ and testing $H_{0}:\theta =0$ versus $H_{0}:\theta \neq 0$ for each method with $n_{1}=500$ and $n_{2}=10\;000$. The invalid IVs $\beta_{j}∼N(0.1,0.1^{2})$ and $\alpha_{j}∼\text{Unif}(0.1,0.2)$ for j=1,…,5 when p=30 and for j=1,…,20 when p=120. For the valid IVs, $\beta_{j}∼N(0.1,0.1^{2})$ when p=30, and $\beta_{j}∼0.5*N(0.1,0.1^{2})$ when p=120. SD, SE, and MSE represent the standard deviation of the estimates, their mean standard error, and mean squared error, respectively.

		θ=0					θ=0.025					θ=0.050				
	Method	Mean	SD	SE	MSE	Type‐I	Mean	SD	SE	MSE	Power	Mean	SD	SE	MSE	Power
p=30	r2SLS‐S	−1.60e−4	0.014	0.013	1.85e−4	0.058	0.025	0.014	0.013	1.90e−4	0.478	0.050	0.014	0.014	2.05e−4	0.960
	r2SLS	0.229	0.037	0.016	0.054	1.000	0.254	0.037	0.017	0.054	1.000	0.279	0.038	0.018	0.054	1.000
	TScML	−1.44e−4	0.013	0.012	1.59e−4	0.054	0.023	0.013	0.012	1.65e−4	0.500	0.046	0.013	0.012	1.87e−4	0.962
	2SLS	0.211	0.028	0.015	0.045	1.000	0.234	0.028	0.016	0.045	1.000	0.258	0.029	0.017	0.044	1.000
p=120	r2SLS‐S	4.91e−4	0.015	0.014	2.23e−4	0.054	0.025	0.015	0.015	2.23e−4	0.414	0.050	0.015	0.015	2.35e−4	0.920
	r2SLS	0.488	0.081	0.027	0.245	1.000	0.514	0.082	0.028	0.245	1.000	0.539	0.082	0.029	0.246	1.000
	TScML	4.69e−4	0.011	0.010	1.15e−4	0.056	0.019	0.011	0.010	1.55e−4	0.468	0.037	0.011	0.010	2.85e−4	0.946
	2SLS	0.360	0.051	0.020	0.132	1.000	0.378	0.051	0.021	0.127	1.000	0.397	0.052	0.022	0.123	1.000

### 3.4 Application 1: r2SLS is More Powerful Than 2SLS in Testing Gene‐Protein Associations

We illustrate the performance of r2SLS‐S against 2SLS via a real data example. Biologically, the genetic information from a gene flows into its encoded protein. Here, we treat the expression of a protein as the outcome and the corresponding gene's expression as the exposure; it is biologically reasonable to assume that they (or at least most of them) are causally associated. We tested $H_{0}:\theta =0$ versus $H_{A}:\theta \neq 0$ for the effect of gene expression on protein expression for each gene‐protein pair. We chose the candidate gene‐protein pairs from two large‐scale Alzheimer's disease (AD) GWASs, IGAP [23] and EADB [24]. Combining their results, we obtained in total of 110 loci significantly associated with AD. For stage 2, we used the latest large‐scale proteomic data from the UK Biobank Pharma Proteomics Project (UKB‐PPP), containing the plasma proteomic profiles of 54 219 participants with 2923 proteins [25]. Among those AD‐associated genes, 24 had their protein expression levels measured in the UKB‐PPP. In addition, 21 of the 24 genes were identified as putative causal AD genes by the Alzheimer's Disease Sequencing Project (ADSP) gene verification committee [26, 27, 28, 29]. We used these 21 genes/proteins for the remaining analysis. The gene expression data for stage 1 were from the GTEx v8 [10]. We used the whole blood gene expression data from 558 participants of European ancestry. We first conducted GWAS for each gene based on the individual‐level gene expression data. For quality control, we only keep the SNPs with a maximum missing rate of 0.05, a minor allele frequency greater than 0.01, and a Hardy‐Weinberg equilibrium test p value above $10^{-6}$. For each gene, we selected its cis‐SNPs from an expanded region of ±500kb surrounding the gene. For one gene, there were no cis‐SNPs from the GTEx v8 data, and it was excluded for further analysis. We further performed clumping with a threshold of $r^{2}=0.5$ to remove highly correlated SNPs. For trans‐SNP selection for each protein, we first removed a small portion of participants with multiple visits and only used the baseline protein measurements in UKB‐PPP. We further removed related individuals to avoid bias from genetic correlations [30], and those from other population/ethnic groups, leaving a final cohort of 38 083 unrelated participants of self‐reported European ancestry for further analysis. We then conducted GWAS for each protein (on its inverse‐normal‐transformed expression levels) with the individual‐level UKB‐PPP data [25], then selected the SNPs with p values $<5*10^{-8}$ after clumping with $r^{2}=0.5$. Among those cis‐SNPs that were available in both GTEx v8 and UKB‐PPP data, we selected the top 50 SNPs with the highest (marginal) absolute correlation to the expression level of the corresponding protein. We used the individual‐level GTEx and UKB‐PPP data in stages 1 and 2, respectively. Since the stage 1 adjusted $R^{2}$ for gene IL34 using both cis‐ and trans‐SNPs was negative, its result was excluded. We also excluded gene SORT1 as its SNP covariance matrix in stage 1 was (nearly) singular.

**FIGURE 2:**
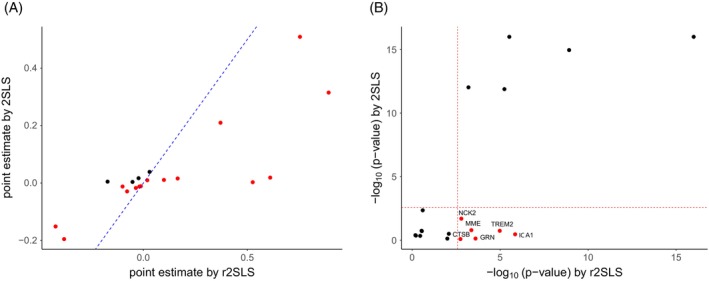
Comparison of r2SLS and 2SLS for inferring associations of 19 AD risk genes' expression levels with their corresponding proteins': (A) The point estimates of θ, where the red dots represent the estimates from r2SLS are larger than those from 2SLS in magnitude and with the same sign, the (blue) line is the identity line; (B) the p values for $H_{0}:\theta =0$, where the red dots represent the eight significant genes identified by r2SLS but not by 2SLS and the black dot represents otherwise, and the (red) lines represent the Bonferroni‐adjusted significance threshold.

We compare the point estimates for each gene and the corresponding p values obtained from r2SLS and 2SLS in Figure 2. The detailed results are provided in Table S3. Figure 2A shows that for the majority, the estimates of the causal effects θ from r2SLS were larger than those from 2SLS in magnitude, confirming the attenuation bias of 2SLS as shown in the simulation study. Figure 2B shows that there were six significant genes (TREM2, MME, CTSB, ICA1, GRN, CTSB) identified by r2SLS, but missed by 2SLS, after the Bonferroni adjustment, indicating that r2SLS is more powerful than 2SLS.

Finally, we also followed the same procedure for trans‐SNP selection for proteins to select trans‐SNPs for each gene with GTEx data. However, only genes CTSH and EPHA1 had any trans‐SNPs selected. By including such trans‐SNPs in 2SLS, we obtained similar results, including their statistical significance levels, though their effect size estimates were larger, as expected. The detailed results are included in Table S4. These results confirmed our main point: Due to a much smaller stage 1 sample size and importantly, expected weak effects of trans‐SNPs, the statistical power would be too low to select trans‐SNPs as IVs in stage 1 for 2SLS or standard TWAS; thus, such a selection is almost always skipped in practice [5, 6]. In contrast, as demonstrated here, with a much larger sample size in stage 2, it is much more feasible with higher power to successfully select some trans‐SNPs; by taking advantage of this feature of genetic data in TWAS, our proposed r2SLS is less biased and more powerful.

### 3.5 Application 2: r2SLS Detects APOE‐AD Association

Due to often small sample sizes of gene expression data, as for GTEx v8, many genes will be excluded from 2SLS‐based TWAS analysis to avoid weak IV bias with their insignificant cis‐heritability (or $R^{2}$) or low F‐statistics (e.g., less than the usual threshold of 10). In this data example, we aimed to show the unnecessary conservativeness of this practice based on 2SLS in the conventional TWAS; in contrast, adopting r2SLS sidesteps this issue. More specifically, we considered a well‐known causal gene APOE for AD. In the GTEx v8 data from the brain hippocampus, which is likely related to AD, there were $n_{1}=150$ samples of European ancestry. With such a small sample size, APOE failed to achieve a significant cis‐heritability (as shown in the Fusion website [6]) or the usual F‐statistic threshold. Hence, in any conventional TWAS analysis, APOE would be excluded. Here, for stage 2, we used the aforementioned IGAP AD GWAS data with 21 982 cases and 41 944 controls. We used the same procedure as in the previous data example to select cis‐SNPs and trans‐SNPs. The reference panel is from those 38 083 UKB‐PPP participants in the previous Section 3.4. But given an even smaller sample size, we chose only 10 IVs as the top 10 cis‐SNPs with the highest absolute correlations to the APOE expression levels for 2SLS, while we obtained 57 additional SNPs significantly associated with AD ($p<5*10^{-8}$) after clumping with $r^{2}=0.5$ (as trans‐SNPs) for r2SLS. For 2SLS, the estimated (causal) effect size of APOE on AD was −0.020, with a standard error of 0.010 and a p value of 0.042. By r2SLS, we obtained a significant p value of 1.68e−4 with a larger (in magnitude) effect estimate of −0.126 and a standard error of 0.035. This example demonstrated again the usefulness of r2SLS in TWAS for picking up a gene missed by 2SLS due to a small sample size in stage 1, yielding higher power and coverage for scientific discoveries.

### 3.6 Application 3: r2SLS Yielded No False Positives in a Negative Control Experiment

Here, we added a real data example to demonstrate the performance of r2SLS for negative controls. We chose a few negative controls for outcome hair color (blonde) with the exposures as high‐density lipoprotein cholesterol (HDL), coronary heart disease (CAD), and asthma. The hair color GWAS is from the Neale Lab (GWAS round 2) with a sample size of 360 270 individuals of European ancestry. The GWASs of the exposures have a sample size of 187 167, 184 305, and 26 475, respectively, from different consortia [31, 32, 33].

With the large sample sizes of the GWAS data, we selected candidate IVs based on the p value threshold of $5*10^{-8}$ in each exposure GWAS for 2SLS with clumping $r^{2}=0.001$. For r2SLS, we selected IVs once based on the hair color GWAS with the same p value and clumping criteria as for 2SLS. The reference panel consisted of those 38 083 UKB‐PPP participants in Section 3.4. We applied both r2SLS and 2SLS and summarized the results in Table 5. The results for both methods remained negative, showing no statistically significant exposure‐outcome associations, thus supporting the use of r2SLS (and 2SLS) in this application.

**TABLE 5:** Inference results of the negative controls of HDL, CAD, asthma for hair color (blonde) for r2SLS and 2SLS.

Exposures	Hair color (outcome)					
	r2SLS			2SLS		
(# of SNPs r2SLS/2SLS)	Effect est	SE	p	Effect est	SE	p
HDL (105/106)	−0.070	0.042	0.09	−0.014	0.008	0.07
CAD (188/35)	−2.48	2.67	0.35	0.006	0.016	0.72
Asthma (42/7)	−0.359	0.213	0.09	0.008	0.015	0.62

## 4 Discussion

We have proposed r2SLS to address some potential limitations of 2SLS, which has been exclusively adopted in many applications for genetic studies, such as current TWAS/PWAS/MWAS. In r2SLS, we draw inference based on an IV‐predicted outcome and an observed exposure, instead of on an IV‐predicted exposure and an observed outcome in the conventional 2SLS. By leveraging often much larger stage 2 samples in TWAS, r2SLS can provide more accurate estimation, whereas 2SLS may suffer from severe attenuation biases, leading to inflated type I errors and loss of power. At the end, the proposed r2SLS can be more powerful than 2SLS, as shown in simulations and two real data applications. In addition, theoretically there is a non‐identifiability issue with 2SLS, but not with r2SLS, if $R^{2}=0$ in the stage 1 model, though, as found in our simulations, numerically it does not seem to be a real problem for hypothesis testing on, but not estimation of, θ with finite samples. This suggests that the current practice of excluding an exposure (e.g., a gene) with a too small $R^{2}$ (or cis‐heritability) from exposure‐outcome association testing in 2SLS/TWAS is overly conservative and unnecessary. In particular, in such a situation (and those with very weakly associated IVs), applying our uncorrected version of r2SLS in Equation (5) performs well for hypothesis testing (but not for parameter estimation), and should be applied. This is highly relevant in practice because with often a small sample size for an exposure, its $R^{2}$ (or cis‐heritability) may be under‐estimated, and thus unnecessarily excluded from TWAS analysis; our proposed method overcomes this severe limitation, as demonstrated in our second data example for APOE‐AD association. Finally, the proposed r2SLS also has a causal interpretation just like 2SLS because of their estimating the same causal parameter [34]; on the other hand, for the same reason, if one would prefer to interpret TWAS as a gene‐based association test for the association between the genetically regulated component of a gene and an outcome, r2SLS can be equally used for this purpose.

One future direction for r2SLS is to further develop and study strategies to deal with invalid IVs, for example, under its own framework, such as under the working model (13). Another direction is to extend the outcome prediction from OLS to other methods, such as penalized or Bayesian regression with high‐dimensional data. Finally, the normality assumption on both the exposure and outcome may be relaxed for large‐sample approximations. These are interesting topics warranting future investigation.

## Conflicts of Interest

The authors declare no conflicts of interest.

## Supporting information




**Data S1.** Supporting Information.

## Data Availability

The data that support the findings of this study are openly available to approved users in the UK Biobank at https://www.ukbiobank.ac.uk/. The R package for r2SLS is available on https://github.com/lei‐fang‐stat/r2SLS. The access to the UKB data and GTEx data was approved through UKB Application #35107 and dbGaP Project #26511, respectively. The hair color (blond), HDL, CAD, and asthma GWAS results can be obtained from *TwoSampleMR* R package with request ID of “ukb‐d‐1747_1”, “ieu‐a‐299”, “ieu‐a‐7”, “ieu‐a‐44”, respectively.
