# Supplementary material for: A Modification to Two‐Stage Least Squares With Genetic Applications
Source: Stat Med. 2025 Nov 7;44(25-27):e70308. doi: 10.1002/sim.70308 (PMC12593333; doi:10.1002/sim.70308)
Supplement: Supplementary file 1 — Data S1. Supporting Information. [file SIM-44-0-s001.pdf]

# A modification to two-stage least squares with genetic applications


Lei Fang

Division of Biostatistics and Health Data Science, University of Minnesota

Wei Pan *

Division of Biostatistics and Health Data Science, University of Minnesota

*Correspondence author: panxx014@umn.edu


## Supplementary Material

The Supplementary Material contains the proof of Theorems 1 and 2, and additional simulation results.

### Proof of Theorem 1

The variance component $(\mathbf{Z}_1\boldsymbol{\beta})^\top\boldsymbol{\varepsilon}\theta/\mathbf{x}_1^\top\mathbf{x}_1$ in $\tilde{\theta}$ has the following distribution:

$$\frac{(\mathbf{Z}_1\boldsymbol{\beta})^\top\boldsymbol{\varepsilon}}{\mathbf{x}_1^\top\mathbf{x}_1}\theta = \frac{\|\mathbf{Z}_1\boldsymbol{\beta}\|_2}{\mathbf{x}_1^\top\mathbf{x}_1}\frac{(\mathbf{Z}_1\boldsymbol{\beta})^\top\boldsymbol{\varepsilon}}{\|\mathbf{Z}_1\boldsymbol{\beta}\|_2}\theta \sim N(0, \theta^2\sigma_\varepsilon^2\|\mathbf{Z}_1\boldsymbol{\beta}\|_2^2/(\mathbf{x}_1^\top\mathbf{x}_1)^2),$$

combine the variance component in (5), it is straightforward to obtain the result in (7). For the asymptotic distribution under $n_1, n_2 \to \infty$ and $\lim_{n_1,n_2\to\infty} n_2/n_1 = \phi \in (0,\infty)$, we notice that $\mathbf{x}_1^\top\mathbf{Z}_1/\mathbf{x}_1^\top\mathbf{x}_1 \to \boldsymbol{\beta}\mathbf{Z}_1^\top\mathbf{Z}_1/\mathbf{x}_1^\top\mathbf{x}_1$ and $(1-\kappa) = (\mathbf{x}_1^\top\mathbf{x}_1 - \boldsymbol{\varepsilon}^\top\boldsymbol{\varepsilon})/\mathbf{x}_1^\top\mathbf{x}_1 \to \boldsymbol{\beta}^\top\Sigma_{\mathbf{Z}}\boldsymbol{\beta}$, combining with the result in (7), we get (8).

### Proof of Theorem 2

The first part of the proof involves the variable selection consistency from TScML. We briefly introduce the method and summarize its main assumptions, but omit its detailed proof.

TScML uses a constrained maximum likelihood approach to select the invalid IVs by optimizing the following function for a given $K$:

$$\hat{\theta}_K, \hat{\boldsymbol{\alpha}}_K = \text{argmin}_{\theta,\boldsymbol{\alpha}}\|Y_2 - \theta\hat{X}_2 - \mathbf{Z}_2\boldsymbol{\alpha}\|^2 \text{ subject to} \frac{1}{\lambda}\sum_{j=1}^{p}\min(|\alpha_j|,\lambda) \le K,$$

where $K$ represents the number of invalid IVs. Then for each $K = 0,\dots,p-2$, TScML uses BIC to determine the optimal $K$ as $\hat{K}$. The $\hat{\boldsymbol{\alpha}}_{\hat{K}}$ identifies the invalid IVs. TScML requires the following two assumptions.

Assumption 1: Assume that $|A \cap B^c| > \max_{c\neq 0}|j \in A : \alpha_j/\beta_j = c|$, where $A$ represents all the candidate IVs for the exposure, and $B$ is defined in the main paper referring to the invalid ones.

Assumption 2: Assume $0 < \lambda \le 1/\sqrt{n_2 \cdot p \cdot c_{\max}(\mathbf{Z}_2^\top\mathbf{Z}_2)}$, where $c_{\max}$ denotes the largest eigenvalue of a matrix.

**Lemma 1**. Assume the covariance matrix of $\mathbf{Z}$ is invertible, and that Assumptions 1 and 2 hold, then as $n_1, n_2 \to \infty$, TScML consistently selects the true set $B$ with $P(\hat{B}_{\hat{K}} = B) \to 1$.

Therefore, after TScML, we treat the selected $\hat{B}$ as the true invalid set $B$. Then for our r2SLS:

$$\begin{aligned}\hat{\theta}' &= \frac{\mathbf{x}_1(\mathbf{I}-\mathbf{P}_{\mathbf{Z}_{1,\hat{B}}})\hat{\mathbf{y}}_1}{\mathbf{x}_1(\mathbf{I}-\mathbf{P}_{\mathbf{Z}_{1,\hat{B}}})\mathbf{x}_1} \\ &= \frac{\mathbf{x}_1(\mathbf{I}-\mathbf{P}_{\mathbf{Z}_{1,\hat{B}}})(\mathbf{x}_1\theta+\mathbf{Z}_1\tilde{\boldsymbol{\gamma}}-\mathbf{Z}_1\boldsymbol{\beta}_1\theta-\mathbf{Z}_{1,\hat{B}}\alpha+\mathbf{Z}_{1,\hat{B}}\alpha-\boldsymbol{\varepsilon}\theta)}{\mathbf{x}_1(\mathbf{I}-\mathbf{P}_{\mathbf{Z}_{1,\hat{B}}})\mathbf{x}_1} \\ &= \theta+\frac{\mathbf{x}_1(\mathbf{I}-\mathbf{P}_{\mathbf{Z}_{1,\hat{B}}})\mathbf{Z}_1(\tilde{\boldsymbol{\gamma}}-\boldsymbol{\gamma})}{\mathbf{x}_1(\mathbf{I}-\mathbf{P}_{\mathbf{Z}_{1,\hat{B}}})\mathbf{x}_1}-\frac{\boldsymbol{\varepsilon}^\intercal(\mathbf{I}-\mathbf{P}_{\mathbf{Z}_{1,\hat{B}}})\boldsymbol{\varepsilon}}{\mathbf{x}_1(\mathbf{I}-\mathbf{P}_{\mathbf{Z}_{1,\hat{B}}})\mathbf{x}_1}\theta-\frac{(\mathbf{Z}_{1,\hat{B}^c}\boldsymbol{\beta}_{\hat{B}^c})^\intercal(\mathbf{I}-\mathbf{P}_{\mathbf{Z}_{1,\hat{B}}})\boldsymbol{\varepsilon}}{\mathbf{x}_1(\mathbf{I}-\mathbf{P}_{\mathbf{Z}_{1,\hat{B}}})\mathbf{x}_1}\theta.\end{aligned}$$

Recall that we define $\kappa' = \frac{\boldsymbol{\varepsilon}^\intercal(\mathbf{I}-\mathbf{P}_{\mathbf{Z}_{1,\hat{B}}})\boldsymbol{\varepsilon}}{\mathbf{x}_1^\intercal(\mathbf{I}-\mathbf{P}_{\mathbf{Z}_{1,\hat{B}}})\mathbf{x}_1}$, $\lim_{n_1\to\infty}\frac{\sqrt{n_1}\|(\mathbf{Z}_{1,\hat{B}^c}\boldsymbol{\beta}_{\hat{B}^c})^\intercal(\mathbf{I}-\mathbf{P}_{\mathbf{Z}_{1,\hat{B}}})\|_2}{\mathbf{x}_1^\intercal(\mathbf{I}-\mathbf{P}_{\mathbf{Z}_{1,\hat{B}}})\mathbf{x}_1} = \tau$, $\lim_{n_1\to\infty}\Phi' = \frac{\mathbf{x}_1^\intercal(\mathbf{I}-\mathbf{P}_{\mathbf{Z}_{1,\hat{B}}})\mathbf{Z}_1}{\mathbf{x}_1^\intercal(\mathbf{I}-\mathbf{P}_{\mathbf{Z}_{1,\hat{B}}})\mathbf{x}_1}$, then we have

$$\begin{aligned}\sqrt{n_2}(\frac{1}{1-\kappa'}\hat{\theta}'-\theta) =& \frac{1}{1-\kappa'}\frac{\mathbf{x}_1^\intercal(\mathbf{I}-\mathbf{P}_{\mathbf{Z}_{1,\hat{B}}})}{\mathbf{x}_1(\mathbf{I}-\mathbf{P}_{\mathbf{Z}_{1,\hat{B}}})\mathbf{x}_1}\sqrt{n_2}(\tilde{\boldsymbol{\gamma}}-\boldsymbol{\gamma})+ \\ & \sqrt{n_2/n_1}\frac{1}{1-\kappa'}\frac{\|(\mathbf{Z}_{1,\hat{B}^c}\boldsymbol{\beta}_{\hat{B}^c})^\intercal(\mathbf{I}-\mathbf{P}_{\mathbf{Z}_{1,\hat{B}}})\|_2}{\mathbf{x}_1(\mathbf{I}-\mathbf{P}_{\mathbf{Z}_{1,\hat{B}}})\mathbf{x}_1}\frac{(\mathbf{Z}_{1,\hat{B}^c}\boldsymbol{\beta}_{\hat{B}^c})^\intercal(\mathbf{I}-\mathbf{P}_{\mathbf{Z}_{1,\hat{B}}})\boldsymbol{\varepsilon}}{\|(\mathbf{Z}_{1,\hat{B}^c}\boldsymbol{\beta}_{\hat{B}^c})^\intercal(\mathbf{I}-\mathbf{P}_{\mathbf{Z}_{1,\hat{B}}})\|_2}\theta \\ & \xrightarrow{d} N\left(0, \frac{1}{(1-\kappa')^2}(\sigma_t^2\Phi'\Psi\Phi'^\intercal+\phi\theta^2\sigma_\varepsilon^2\tau^2)\right).\end{aligned}$$

## Proof of Theorem 3

We rewrite the full model for the multivariate case.

$$\begin{cases}\mathbf{x}_{1,1} = \mathbf{Z}_1\boldsymbol{\beta}_1+\boldsymbol{\varepsilon}_{\mathbf{x_1}} \\ \vdots \\ \mathbf{x}_{1,d} = \mathbf{Z}_1\boldsymbol{\beta}_d+\boldsymbol{\varepsilon}_{\mathbf{x_d}} \\ \mathbf{y}_2 = \mathbf{x}_1\theta_1+\cdots+\mathbf{x}_d\theta_d+\boldsymbol{\varepsilon}_{\mathbf{y}}.\end{cases}$$

Let use $\mathbf{X}$ denote $\{\mathbf{x}_{1,1},\cdots,\mathbf{x}_{1,d}\}$, and $\boldsymbol{\theta} = \{\theta_1,\cdots,\theta_d\}^\intercal$,

$$\begin{aligned}\widehat{\boldsymbol{\theta}} &= (\mathbf{X}^\intercal\mathbf{X})^{-1}\mathbf{X}\widehat{\boldsymbol{y}}_1 \\ &= (\mathbf{X}^\intercal\mathbf{X})^{-1}\mathbf{X}^\intercal(\mathbf{Z}_1\hat{\boldsymbol{\gamma}}) \\ &= (\mathbf{X}^\intercal\mathbf{X})^{-1}\mathbf{X}^\intercal(\mathbf{Z}_1\hat{\boldsymbol{\gamma}}+\mathbf{X}\boldsymbol{\theta}-\mathbf{X}\boldsymbol{\theta}) \\ &= (\mathbf{X}^\intercal\mathbf{X})^{-1}\mathbf{X}^\intercal(\mathbf{X}\boldsymbol{\theta}+\mathbf{Z}_1(\hat{\boldsymbol{\gamma}}-\boldsymbol{\gamma})-\sum_{j=1}^d\boldsymbol{\varepsilon}_{\mathbf{x_j}}\theta_j) \\ &= \boldsymbol{\theta}+(\mathbf{X}^\intercal\mathbf{X})^{-1}\mathbf{X}^\intercal\mathbf{Z}_1(\hat{\boldsymbol{\gamma}}-\boldsymbol{\gamma})-(\mathbf{X}^\intercal\mathbf{X})^{-1}\mathbf{X}^\intercal\sum_{j=1}^d\boldsymbol{\varepsilon}_{\mathbf{x_j}}\theta_j\end{aligned}$$

$$\mathbf{X}^\intercal\sum_{j=1}^d\boldsymbol{\varepsilon}_{\mathbf{x_j}}\theta_j = \begin{bmatrix}(\mathbf{Z}_1\boldsymbol{\beta}_1)^\intercal\sum_{j=1}^d\boldsymbol{\varepsilon}_{\mathbf{x_j}}\theta_j \\ \vdots \\ (\mathbf{Z}_1\boldsymbol{\beta}_d)^\intercal\sum_{j=1}^d\boldsymbol{\varepsilon}_{\mathbf{x_j}}\theta_j\end{bmatrix}+\begin{bmatrix}(\boldsymbol{\varepsilon}_{\mathbf{x_1}})^\intercal\sum_{j=1}^d\boldsymbol{\varepsilon}_{\mathbf{x_j}}\theta_j \\ \vdots \\ (\boldsymbol{\varepsilon}_{\mathbf{x_d}})^\intercal\sum_{j=1}^d\boldsymbol{\varepsilon}_{\mathbf{x_j}}\theta_j\end{bmatrix}$$

Since $\{\boldsymbol{\varepsilon}_{x_1}, \cdots, \boldsymbol{\varepsilon}_{x_d}\}$ follows multivariate normal distribution, we have $\sum_{j=1}^{d} \boldsymbol{\varepsilon}_{\mathbf{x_j}}\theta_j \sim N(0, \sigma_{tX}^2)$.

Since $\sqrt{n_2}(\hat{\boldsymbol{\gamma}} - \boldsymbol{\gamma}) \xrightarrow{d} N(0, \sigma_t^2 E(\mathbf{Z}_1^\top \mathbf{Z}_1)^{-1})$, where $\sigma_t^2$ is the total variance of $\sum_{j=1}^{d} \boldsymbol{\varepsilon}_{\mathbf{x_j}}\theta_j + \boldsymbol{\varepsilon}_{\mathbf{y}}$, we have

$$\sqrt{n_2}(\mathbf{X}^\top\mathbf{X})^{-1}\mathbf{X}^\top\mathbf{Z}_1(\hat{\boldsymbol{\gamma}} - \boldsymbol{\gamma}) \xrightarrow{d} MN(\mathbf{0}, \mathbf{W}^\top\boldsymbol{\Omega}\mathbf{W}),$$

where $\mathbf{W}$ represents $\text{limit}_{n_1\to\infty}(\mathbf{X}^\top\mathbf{X})^{-1}\mathbf{X}^\top\mathbf{Z}_1$ and $\boldsymbol{\Omega}$ represents $\sigma_t^2 E(\mathbf{Z}_1^\top \mathbf{Z}_1)^{-1}$.

For the second error component, firstly, we have

$\sqrt{n_2}(\mathbf{X}^\top\mathbf{X})^{-1} \begin{bmatrix} (\mathbf{Z}_1\boldsymbol{\beta}_1)^\top \sum_{j=1}^{d} \boldsymbol{\varepsilon}_{\mathbf{x_j}}\theta_j \\ \vdots \\ (\mathbf{Z}_1\boldsymbol{\beta}_d)^\top \sum_{j=1}^{d} \boldsymbol{\varepsilon}_{\mathbf{x_j}}\theta_j \end{bmatrix} \xrightarrow{d} MN(0, \frac{n_2}{n_1}\sigma_{tX}^2 (\Sigma_{\mathbf{X}})^{-1}\boldsymbol{\beta}^\top\Sigma_{\mathbf{Z}_1}\boldsymbol{\beta}(\Sigma_{\mathbf{X}})^{-1})$, Let $\mathbf{Z}_1\boldsymbol{\beta}$ denotes $(\mathbf{Z}_1\boldsymbol{\beta}_1, \cdots, \mathbf{Z}_1\boldsymbol{\beta}_d)$, we have

$$\begin{aligned} E\left(\begin{bmatrix}\hat{\theta}_1\\ \vdots \\ \hat{\theta}_d\end{bmatrix}\right) &= \left(\begin{bmatrix}\theta_1\\ \vdots \\ \theta_d\end{bmatrix}\right) - E\left((\mathbf{X}^\top\mathbf{X})^{-1}\begin{bmatrix}(\boldsymbol{\varepsilon}_{\mathbf{x_1}})^\top \sum_{j=1}^{d}\boldsymbol{\varepsilon}_{\mathbf{x_j}}\theta_j \\ \vdots \\ (\boldsymbol{\varepsilon}_{\mathbf{x_d}})^\top \sum_{j=1}^{d}\boldsymbol{\varepsilon}_{\mathbf{x_j}}\theta_j\end{bmatrix}\right) \\ &= \left\{\mathbf{I}_d - E\left((\mathbf{X}^\top\mathbf{X})^{-1}\begin{bmatrix}(\boldsymbol{\varepsilon}_{\mathbf{x_1}})^\top\boldsymbol{\varepsilon}_{\mathbf{x_1}} & \cdots & (\boldsymbol{\varepsilon}_{\mathbf{x_1}})^\top\boldsymbol{\varepsilon}_{\mathbf{x_d}} \\ \vdots & \vdots & \vdots \\ (\boldsymbol{\varepsilon}_{\mathbf{x_d}})^\top\boldsymbol{\varepsilon}_{\mathbf{x_1}} & \cdots & (\boldsymbol{\varepsilon}_{\mathbf{x_d}})^\top\boldsymbol{\varepsilon}_{\mathbf{x_d}}\end{bmatrix}\right)\right\}\begin{bmatrix}\theta_1\\ \vdots \\ \theta_d\end{bmatrix} \end{aligned}$$

therefore to get the unbiased estimator, we have

$$\left\{\mathbf{I}_d - E\left((\mathbf{X}^\top\mathbf{X})^{-1}\begin{bmatrix}(\boldsymbol{\varepsilon}_{\mathbf{x_1}})^\top\boldsymbol{\varepsilon}_{\mathbf{x_1}} & \cdots & (\boldsymbol{\varepsilon}_{\mathbf{x_1}})^\top\boldsymbol{\varepsilon}_{\mathbf{x_d}} \\ \vdots & \vdots & \vdots \\ (\boldsymbol{\varepsilon}_{\mathbf{x_d}})^\top\boldsymbol{\varepsilon}_{\mathbf{x_1}} & \cdots & (\boldsymbol{\varepsilon}_{\mathbf{x_d}})^\top\boldsymbol{\varepsilon}_{\mathbf{x_d}}\end{bmatrix}\right)\right\}^{-1} E\left(\begin{bmatrix}\hat{\theta}_1\\ \vdots \\ \hat{\theta}_d\end{bmatrix}\right) = \begin{bmatrix}\theta_1\\ \vdots \\ \theta_d\end{bmatrix}$$

The correcting factor for the multivariate case is hence

$\Lambda = \left\{\mathbf{I}_d - E\left((\mathbf{X}^\top\mathbf{X})^{-1}\begin{bmatrix}(\boldsymbol{\varepsilon}_{\mathbf{x_1}})^\top\boldsymbol{\varepsilon}_{\mathbf{x_1}} & \cdots & (\boldsymbol{\varepsilon}_{\mathbf{x_1}})^\top\boldsymbol{\varepsilon}_{\mathbf{x_d}} \\ \vdots & \vdots & \vdots \\ (\boldsymbol{\varepsilon}_{\mathbf{x_d}})^\top\boldsymbol{\varepsilon}_{\mathbf{x_1}} & \cdots & (\boldsymbol{\varepsilon}_{\mathbf{x_d}})^\top\boldsymbol{\varepsilon}_{\mathbf{x_d}}\end{bmatrix}\right)\right\}^{-1}$, which can be estimated via $\left\{\mathbf{I}_d - E\left({\Sigma_X}^{-1}\Sigma_{\varepsilon}\right)\right\}^{-1}$, where $\Sigma_X$ is the covariance matrix of $\mathbf{X}$, and each entry in $\Sigma_{\varepsilon}$ is the covariance of $\boldsymbol{\varepsilon}_{\mathbf{x_i}}$ and $\boldsymbol{\varepsilon}_{\mathbf{x_j}}$.

## Additional Numerical results

| | methods | $\theta$=0 | | | | | $\theta$=0.1 | | | | | $\theta$=0.2 | | | | |
|---|---|---|---|---|---|---|---|---|---|---|---|---|---|---|---|---|
| | | mean | SD | SE | MSE | Type I | mean | SD | SE | MSE | Power | mean | SD | SE | MSE | Power |
| $p = 30$ | r2SLS-S1S2(Olkin-Pratt) | 1.26e-4 | 0.020 | 0.021 | 4.01e-4 | 0.042 | 0.102 | 0.022 | 0.023 | 4.96e-4 | 0.996 | 0.204 | 0.028 | 0.028 | 8.08e-4 | 1.000 |
| $\beta_j \sim N(0.1, 0.1^2)$ | r2SLS-S1S2(naive) | 1.32e-4 | 0.020 | 0.021 | 4.03e-4 | 0.042 | 0.102 | 0.022 | 0.023 | 5.03e-4 | 0.996 | 0.204 | 0.028 | 0.028 | 8.29e-4 | 1.000 |
| | r2SLS-S1S2(mle) | 1.47e-4 | 0.017 | 0.017 | 2.76e-4 | 0.042 | 0.085 | 0.018 | 0.019 | 5.45e-4 | 0.996 | 0.169 | 0.021 | 0.022 | 1.37e-3 | 1.000 |
| $p = 120$ | r2SLS-S1S2(Olkin-Pratt) | 1.21e-3 | 0.027 | 0.027 | 7.32e-4 | 0.042 | 0.104 | 0.031 | 0.029 | 9.55e-4 | 0.972 | 0.207 | 0.041 | 0.033 | 1.71e-3 | 1.000 |
| $\beta_j \sim 0.5 * N(0.1, 0.1^2)$ | r2SLS-S1S2(adjusted) | 1.21e-3 | 0.027 | 0.027 | 7.35e-4 | 0.042 | 0.104 | 0.031 | 0.029 | 9.60e-4 | 0.972 | 0.207 | 0.041 | 0.033 | 1.72e-3 | 1.000 |
| | r2SLS-S1S2(unadjusted) | 8.28e-4 | 0.014 | 0.015 | 2.09e-4 | 0.042 | 0.057 | 0.015 | 0.015 | 2.04e-3 | 0.972 | 0.114 | 0.017 | 0.017 | 7.68e-3 | 1.000 |

Table S1: Simulation results for estimating $\theta$ and testing $H_0 : \theta_1^0 = 0$ versus $H_0 : \theta_1^0 \neq 0$ for r2SLS using different methods to estimate $R^2$ with $n_1 = 500$, $n_2 = 10000$; SD, SE, MSE represent the standard deviation of the estimates, their mean standard error, and mean squared error, respectively.



| | methods | $\theta = 0$ | | | | | $\theta = 50$ | | | | |
|---|---|---|---|---|---|---|---|---|---|---|---|
| | | mean | SD | SE | MSE | Type I | mean | SD | SE | MSE | Power |
| $p = 30$ | 2SLS | 0.001 | 0.037 | 0.038 | 1.37e-3 | 0.034 | 8.035 | 3.641 | 2.427 | 1774.303 | 0.860 |
| $\beta_j \sim N(0.02, 0.02^2)$ | r2SLS(without-scaling) | 1.72e-4 | 2.48e-3 | 2.61e-3 | 6.18e-6 | 0.048 | 0.584 | 0.283 | 0.131 | 2442.035 | 0.886 |
| true $R^2 = 0.012$ | r2SLS-naive | 1.72e-4 | 2.48e-3 | 2.40e-3 | 6.18e-6 | 0.058 | 0.584 | 0.283 | 0.270 | 2442.035 | 0.566 |
| | observed-1 | 0.245 | 0.041 | 0.043 | 0.062 | 1.000 | 50.245 | 0.041 | 0.043 | 0.062 | 1.000 |
| | observed-2 | 0.246 | 0.010 | 0.010 | 0.061 | 1.000 | 50.246 | 0.010 | 0.010 | 0.061 | 1.000 |
| $p = 30$ | 2SLS | 0.002 | 0.040 | 0.042 | 0.0016 | 0.028 | 0.057 | 2.010 | 2.048 | 2498.369 | 0.024 |
| $\beta_j = 0$ | r2SLS (without-scaling) | 1.95e-4 | 0.002 | 0.002 | 5.06e-6 | 0.038 | 2.15e-4 | 0.118 | 0.121 | 2499.993 | 0.046 |
| true $R^2 = 0$ | r2SLS-naive | 1.95e-4 | 0.002 | 0.002 | 5.06e-6 | 0.034 | 2.15e-4 | 0.118 | 0.122 | 2499.993 | 0.050 |
| | observed-1 | 0.248 | 0.041 | 0.043 | 0.063 | 1.000 | 50.25 | 0.041 | 0.043 | 0.063 | 1.000 |
| | observed-2 | 0.249 | 0.010 | 0.010 | 0.062 | 1.000 | 50.25 | 0.010 | 0.010 | 0.062 | 1.000 |

Table S2: Simulation results for estimating $\theta$ and testing $H_0 : \theta = 0$ versus $H_0 : \theta \neq 0$ for each method with $n_1 = 500$, $n_2 = 10000$; SD, SE, MSE represent the standard deviation of the estimates, their mean standard error, and mean squared error, respectively. r2SLS(without-scaling) utilized the equation (5) for inference.

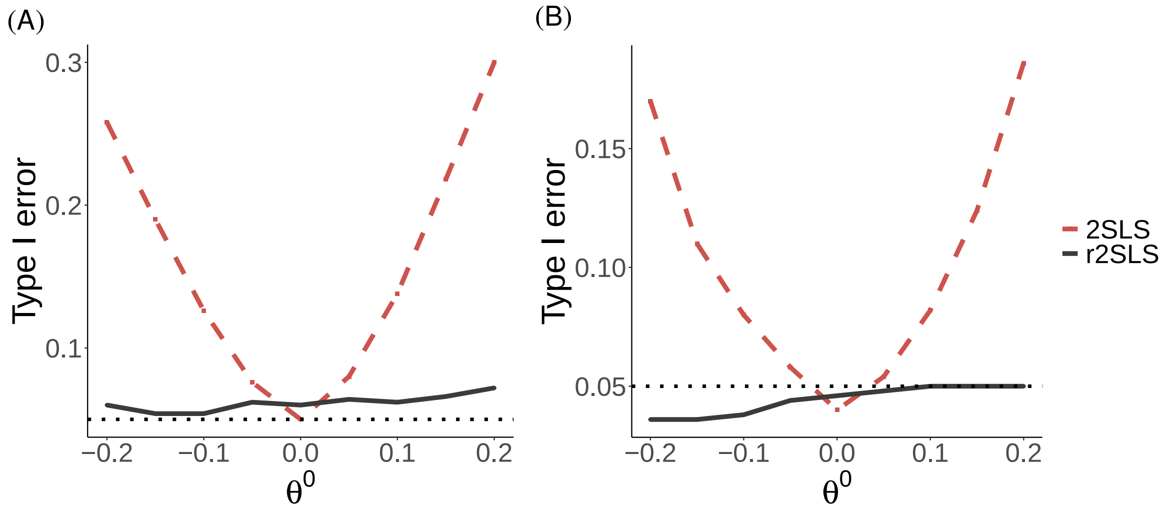


Figure S1: Comparison of r2SLS and 2SLS in simulations: (A) Type I errors for r2SLS and 2SLS for testing $H_0 : \theta = \theta^0$ versus $H_A : \theta \neq \theta^0$ with 30 cis-SNPs and with the outcome following a $t_5$-distribution; (B) similar to (A) but with the outcome following an exponential distribution exp(0.5).

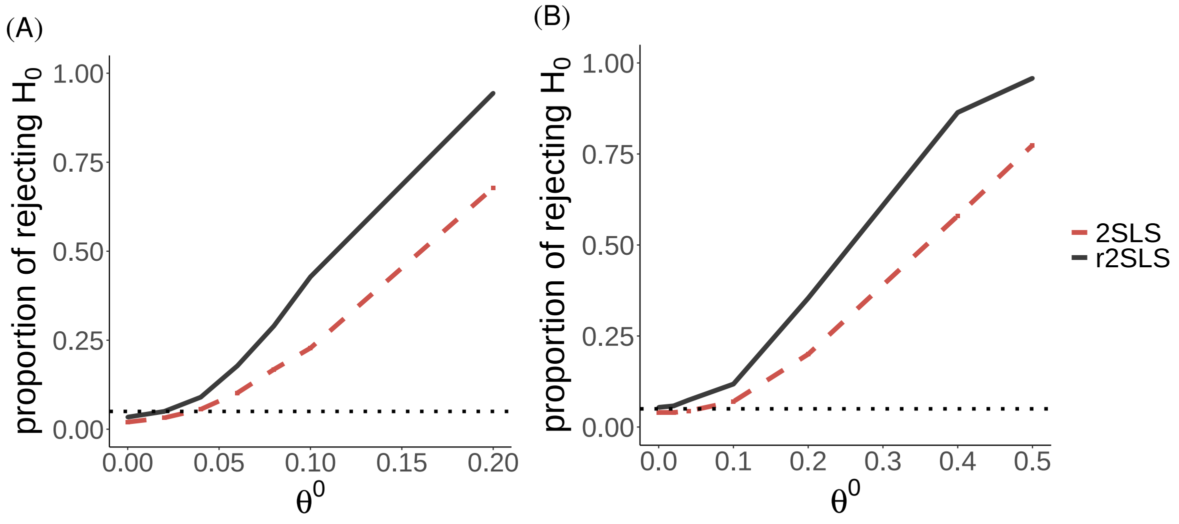


Figure S2: Comparison of r2SLS and 2SLS in simulations: (A) Type I error and power for r2SLS and 2SLS for testing $H_0 : \theta = \theta^0$ versus $H_A : \theta \neq \theta^0$ with additional 20 trans-SNPs with $\beta_j \sim N(0.05, 0.1^2)$ for $j = 21, \dots, 40$ and the outcome follows a $t_5$-distribution; (B) similar to (A) but with the outcome following an exponential distribution exp(0.5).

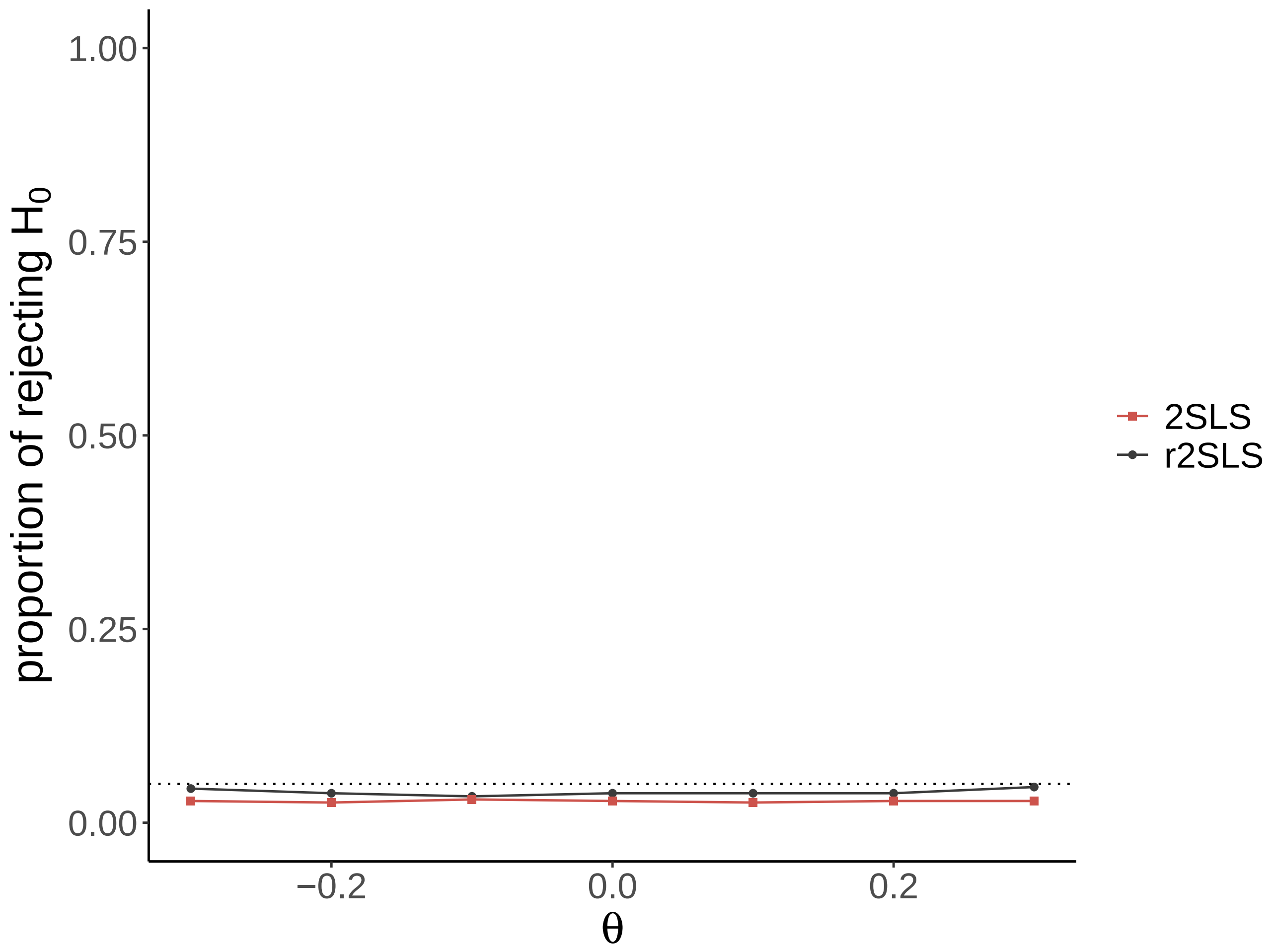


Figure S3: Comparison of r2SLS and 2SLS for testing $H_0 : \theta = 0$ versue $H_A : \theta \neq 0$ in the simulation where $\beta_j = 0$ and $\alpha_j = 0$ for $j = 1, \ldots, 30$ and IVs are generated with autocorrelation of 0.5.

| protein | GTEx | | | | | |
|---|---|---|---|---|---|---|
| | 2SLS (50 cis-SNPs) | | | r2SLS (50 cis-SNPs+trans-SNPs) | | |
| (# of cis+trans SNPs) | effect est | SE | p-value | effect est | SE | p-value |
| TREM2 (130) | 0.016 | 0.012 | 0.18 | 0.165 | 0.037 | 1.0e-5 |
| ICA1 (70) | 0.011 | 0.011 | 0.34 | 0.099 | 0.021 | 1.4e-6 |
| MME (151) | 0.019 | 0.013 | 0.16 | 0.610 | 0.173 | 4.3e-4 |
| GRN (170) | 0.005 | 0.013 | 0.73 | -0.172 | 0.047 | 2.5e-4 |
| NCK2 (54) | -0.029 | 0.012 | 0.02 | -0.078 | 0.025 | 1.6e-3 |
| CTSB (446) | 0.003 | 0.012 | 0.80 | 0.526 | 0.169 | 1.8e-3 |
| CTSH (117) | 0.509 | 0.042 | 0 | 0.753 | 0.078 | 0 |
| CR1 (143) | 0.210 | 0.026 | 1.1e-15 | 0.371 | 0.651 | 1.2e-9 |
| APOE (119) | -0.195 | 0.023 | 0 | -0.381 | 0.082 | 3.0e-6 |
| TREML2 (113) | -0.151 | 0.021 | 1.3e-12 | -0.422 | 0.093 | 5.7e-6 |
| ACE (98) | 0.315 | 0.044 | 9.4e-13 | 0.891 | 0.261 | 6.3e-4 |
| APP (112) | -0.012 | 0.012 | 0.31 | -0.100 | 0.038 | 8.2e-3 |
| EPHA1 (77) | 0.004 | 0.012 | 0.74 | -0.052 | 0.021 | 0.01 |
| CD2AP (85) | -0.017 | 0.013 | 0.19 | -0.036 | 0.034 | 0.28 |
| CLU (59) | 0.039 | 0.014 | 4.4e-3 | 0.030 | 0.026 | 0.25 |
| INPP5D (58) | 0.017 | 0.012 | 0.18 | -0.023 | 0.022 | 0.29 |
| ADAMTS1 (50) | 0.010 | 0.013 | 0.45 | 0.019 | 0.020 | 0.35 |
| BLNK (68) | -0.012 | 0.015 | 0.43 | -0.019 | 0.038 | 0.61 |
| TNIP1 (55) | -0.011 | 0.013 | 0.40 | -0.012 | 0.026 | 0.65 |

Table S3: Inference results of the association between each AD risk gene's expression and its corresponding protein expression using 2SLS and r2SLS with the GTEx and UKB-PPP data.

| protein | GTEx | | | | | |
|---|---|---|---|---|---|---|
| | 2SLS (50 cis-SNPs) | | | 2SLS (50 cis-SNPs+trans-SNPs) | | |
| (# of cis+trans SNPs) | effect est | SE | p-value | effect est | SE | p-value |
| CTSH (52) | 0.336 | 0.031 | 0 | 0.509 | 0.042 | 0 |
| EPHA1 (52) | 0.004 | 0.012 | 0.74 | -0.003 | 0.011 | 0.81 |

Table S4: Inference results of the association between CTSH and EPHA1 and their corresponding protein expression for 2SLS using cis-SNPs only, and cis-SNPs and trans-SNPs(selected based on GTEx data) with the GTEx and UKB-PPP data.
